# Endovascular Treatment of Hydrocephalus: A Systematic Literature Review

**DOI:** 10.1007/s00062-026-01614-y

**Published:** 2026-01-21

**Authors:** Olga Ciobanu-Caraus, Philip Heesen, Nicolin Hainc, Markus A. Möhlenbruch, Martin Bendszus

**Affiliations:** 1 Department of Neuroradiology, Heidelberg University, Heidelberg, Germany; 2 Department of Biostatistics at the Epidemiology, Biostatistics and Prevention Institute, University of Zurich, Zurich, Switzerland; 3 Department of Neuroradiology, University of Zurich, Zurich, Switzerland

**Keywords:** Endovascular treatment, Hydrocephalus, EShunt, Shunt, Neurointervention

## Abstract

**Introduction:**

Hydrocephalus is traditionally managed with ventriculoperitoneal shunting (VPS), which is associated with high rates of infection, mechanical failure, and surgical revisions. Recent innovations in endovascular techniques have led to the development of minimally invasive cerebrospinal fluid (CSF) diversion techniques. This literature review systematically examines the development, procedural techniques, efficacy, and safety profile of endovascular treatments for communicating hydrocephalus.

**Methods:**

A systematic search was conducted in PubMed/MEDLINE, Embase, Scopus and clinicaltrials.gov from inception until March 15, 2025, following the PRISMA guidelines (PROSPERO registration number: CRD420251012455). Two reviewers independently screened and extracted data. Results are summarized descriptively.

**Results:**

Of 5319 records identified, 16 studies were included. Of those, six were published as original articles and 10 as conference abstracts. Overall, two technical reports of endovascular treatment of hydrocephalus were found: eShunt® (*n* = 14 studies) and choroidal artery embolization (*n* = 1 studies). Outcome data are available for 70 patients treated with the eShunt®. In patients with idiopathic normal pressure hydrocephalus treated with eShunt® implantation, the Timed Up and Go test improved by 35.7%, Montreal Cognitive Assessment scores by +1.6 points, and neurogenic bladder symptom scores by −3.6s at one year. No device-related serious adverse events were reported.

**Discussion:**

Endovascular CSF diversion represents an emerging, minimally invasive alternative to traditional shunting techniques for communicating hydrocephalus. While early results demonstrate technical feasibility and encouraging safety profiles, long-term data from ongoing clinical trials are critical to establish its role in standard neurosurgical and neurointerventional practice.

**Supplementary Information:**

The online version of this article (10.1007/s00062-026-01614-y) contains supplementary material, which is available to authorized users.

## Introduction

Hydrocephalus is a common neurological condition affecting approximately 85 per 100.000 individuals, with an increasing prevalence in the elderly population [1]. It is characterized by the pathological accumulation of cerebrospinal fluid (CSF) within the cerebral ventricles, often resulting in increased intracranial pressure and associated neurological deficits [2]. Hydrocephalus may be caused by a broad spectrum of underlying disorders such as subarachnoid hemorrhage [3]. Normal pressure hydrocephalus is a form of communicating hydrocephalus, marked by ventricular enlargement without sustained elevation of intracranial pressure and clinically characterized by the Hakim triad of gait disturbance, cognitive decline, and urinary incontinence [4]. The current standard treatment represents surgical diversion of CSF, most commonly via ventriculoperitoneal shunting (VPS) [5].

While VPS has been the standard of care for decades, they are associated with significant complication rates, including infections, mechanical failures, overdrainage, and the need for frequent revisions [6–8].

Latest advancements in endovascular technology have led to the development of minimally invasive CSF shunt systems, which aim to replicate the natural function of arachnoid granulations by facilitating passive CSF absorption into the venous system [9]. Recently, several case reports have assessed the safety and efficacy of endovascular shunting, and prospective trials have been initiated.

However, no comprehensive review has systematically analyzed the existing literature on endovascular treatment of hydrocephalus, encompassing its mechanism of action, procedural techniques, efficacy, and safety profile. This systematic literature review aims to explore the historical development, technical aspects, current evidence and prospects of endovascular treatment of communicating hydrocephalus.

## Methods

### Protocol and Guidance

This systematic literature review was carried out according to the Preferred Reporting Items for Systematic Reviews and Meta-Analyses (PRISMA) guideline. The protocol for this study was registered on the International Prospective Register of Systematic Reviews (PROSPERO; registration number CRD420251012455).

### Search Strategy and Study Selection

PubMed/MEDLINE, Embase, and Scopus databases were queried to identify eligible studies from inception until March 15th, 2025. The following search strategy was used: (endovascular[Title/Abstract]) AND (shunt[Title/Abstract] OR hydrocephalus[Title/Abstract]). The detailed search strategy structured for each database is available in Supplementary Table 1. References of included studies were hand-searched for eligible articles. In addition, clinicaltrials.gov was searched for currently ongoing clinical trials. Eligible abstracts were imported into Covidence Review Manager. Duplicate articles were excluded automatically. Two independent reviewers (O.C.C., P.H.) performed title and abstract screening and full-text review in duplicate. Any disagreements were resolved by discussion and consensus.

### Eligibility Criteria

Eligible studies include randomized controlled trials (RCTs), non-randomized studies, cohort studies, case series, case reports, preclinical (animal/in vitro) studies, feasibility trials and conference abstracts. Only articles in English were considered. We included studies reporting on endovascular procedures in adult or pediatric patients aimed at CSF diversion, including but not limited to eShunt® systems or related neurointerventional techniques in adult or pediatric patients with hydrocephalus.

### Data Collection

Data were extracted in duplicate by two independent reviewers (O.C.C., P.H.). Extracted variables included study design, sample size, patient demographics, intervention details, and—if available—outcomes and adverse events.

## Results

### Literature Search and Study Selection

**Fig. 1:**
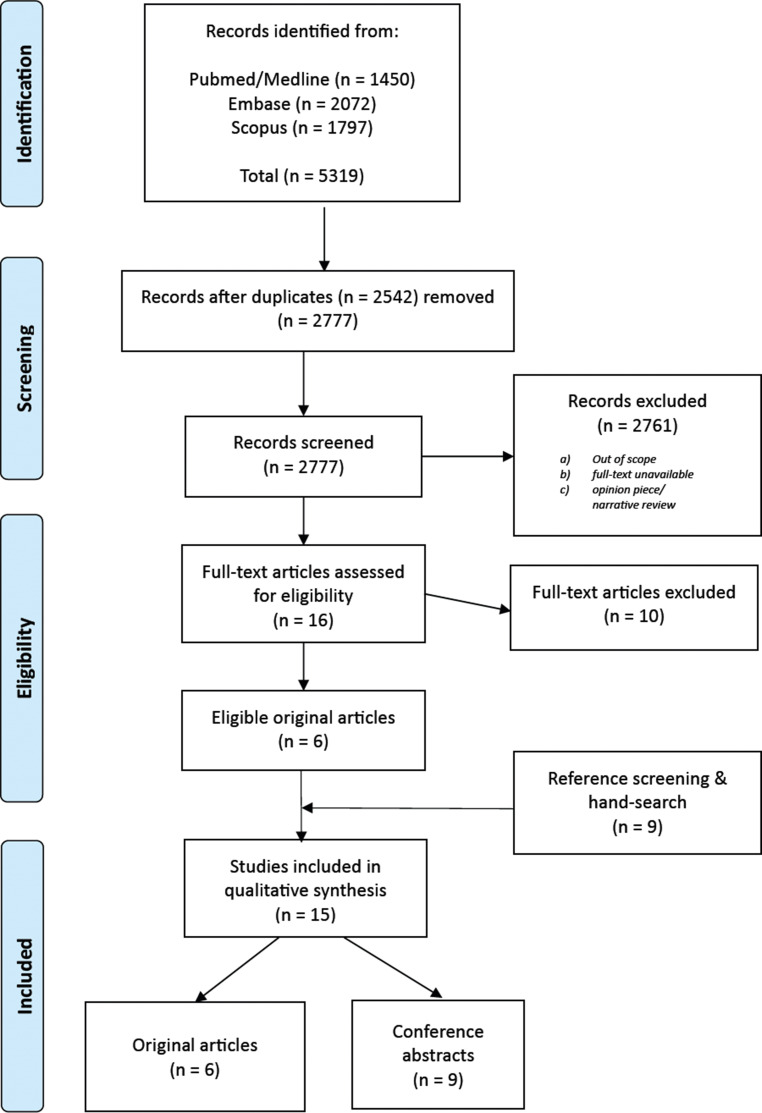
PRISMA Flow chart.

A PRISMA flowchart is given in Fig. 1. A total of 5319 records were identified through database searches. After deduplication, 2777 studies remained for screening. Following title and abstract screening, full-text review and reference screening, a total of six original articles [10–15] and 9 conference abstracts [9, 16–23] were identified. The reasons for exclusion are detailed in Supplementary Table 2. Of 16 included articles, fourteen describe the technical details and outcomes of the eShunt® system [9, 10, 12–23]. One study is a case report of a pediatric patient undergoing successful choroidal artery embolization in hydrocephalus caused by diffuse villous hyperplasia of the choroid plexus (DVHCP) [11].

### Study Characteristics

The characteristics of the original peer-reviewed articles are detailed in Table 1. Of these six articles, two were retrospective morphometric magnetic resonance imaging (MRI) studies [10, 14] and four were case reports/technical reports [11–13, 15]. The studies were published between 2018 and 2025 and were conducted primarily in the USA and Argentina. Subject types included humans (*n* = 16), cadaveric or MRI-based anatomical or technical feasibility studies (*n* = 3). The characteristics of conference abstracts are presented in Table 2.

**Table 1:** Characteristics of included studies sorted by year of publication.

Author et al. [Year]	Title	Country	Study Type/Design	Study population	Type of Hydrocephalus	Main Findings
Heilman et al. [15]	Anatomical characterization of the inferior petrosal sinus and adjacent cerebellopontine angle cistern for development of an endovascular transdural cerebrospinal fluid shunt	USA	Retrospective anatomical MRI study	36 adults	Not applicable (healthy subjects)	Analysis of dimension of the IPS, CPA cistern and distances to adjacent neurovascular structures. Feasibility of targeting IPS and CPA cistern for endovascular shunt in adults
Li et al. [16]	Choroidal artery embolization in the management of cerebrospinal fluid overproduction: case report and review of the literature	USA	Case report	1 child	Communicating, Diffuse villous hyperplasia of the choroid plexus (DVHCP)	Anterior choroidal artery embolization may be a successful adjuvant treatment for CSF overproduction due to DVHCP
Lylyk et al. [17]	First-in-human endovascular treatment of idiopathic intracranial hypertension using a miniature biomimetic transdural shunt	Argentina, USA	Case report	1 adult	Communicating, post-SAH	First successful in-human endovascular eShunt® implant in post-SAH hydrocephalus; rapid ICP reduction and ventriculomegaly resolution
Malek et al. [18]	Endovascular Shunting for Communicating Hydrocephalus Using a Biologically Inspired Transdural Cerebrospinal Fluid Valved eShunt® Implant	USA, Argentina	Technical report	Not applicable (review + pilot data)	Communicating, NPH	Description of eShunt® rationale and design; pilot data show rapid ICP normalization in hydrocephalus. Early results indicate lower infection/malfunction risk than VPS; ongoing trials for hydrocephalus, NPH, IIH
Colasurdo et al. [19]	Morphometric analysis of the inferior petrosal sinus and cerebellopontine angle cistern to assess feasibility of endovascular shunting in pediatric patients	USA	Retrospective morphometric MRI study	100 children	Not applicable (healthy subjects)	Morphometric analysis shows most children > 1 year meet IPS and CPA anatomical criteria for eShunt® implantation
Koo et al. [9]	eShunt® system: a technical video	USA, Argentina	Technical video/procedural description	Not applicable	Communicating	Patient selection and procedural details for eShunt® system deployment in communicating hydrocephalus

*CPA* cerebellopontine angle; *CSF* cerebrospinal fluid; *DVHCP* diffuse villous hyperplasia of the choroid plexus; *ICP* intracranial pressure; *IIH* idiopathic intracranial hypertension; *IPS* inferior petrosal sinus; *MRI* magnetic resonance imaging; *NPH* normal pressure hydrocephalus; *SAH* subarachnoid hemorrhage; *VPS* ventriculoperitoneal shunt

**Table 2:** Study characteristics of published conference abstracts.

Author et al. [Year]	Country	Study Design	Type of Hydrocephalus	Study population			Results						
				Treated	F	Mean age ± SD (yrs)	EVD removal by 36–48 hrs post insertion	ICP decrease	Improvement in				SAE
									NPH sx	TUG	MOCA	NBSS	
Lylyk et al. (O-038) [20]	Argentina, USA	PO	Post-aSAH	7	5	64.7 ± 12	7/7 (100%)	At 1 hr: from 33.4 to 13 cmH2O at 36 hrs: to 9 cmH2O	NR	NR	NR	NR	No delayed hemorrhage
Lylyk et al. (O-039) [21]	Argentina, USA	PO	iNPH	11	4	74.87 ± 4.2	NR	NR	NR	At 30 days: 35.4%* At 90 days: 24.8%* at 180 days: 32.8%*	At 30 days:*	At 30-, 90- and 180-days:*	NR
Lylyk et al. (O14) [22]	Argentina, USA	PO	–	14	–	–	NR	NR	NR	NR	NR	NR	One-year safety: no radiologic or symptomatic overdrainage, hemorrhage, or implant migration. mRS stable/improved to baseline
			NPH	9	4	75.7 ± 7.3	–	–	–	–	–	–	
			Post-aSAH	4	3	57.5 ± 16.5	–	–	–	–	–	–	
			IIH	1	0	50	–	–	–	–	–	–	
Lylyk et al. (O-037) [23]	Argentina, USA	PO	NPH	9	4	75.7 ± 7.3	NR	NR	NR	At 1 yr: 35.7%*	At 1 yr: +1.6 points*	At 1 yr: –3.6 points	NR
Lylyk et al. (P10–4.014) [9]	Argentina, USA	PO	iNPH	19	7	75.2 ± 8.8	NR	NR	15/16 (93.8%)	At 30 days: 32.1%* At 90 days: 33.3%* At 180-days: 39.0%*	NR	NR	NR
Lylyk et al. (S16.004) [20]	Argentina, USA	PO	Post-aSAH	9	6	62.6 ± 21.4	9/9 (100%)	At 1 hr: from 34.4 to 10.8 cm H2O* at 36 hrs: 10.9 cm H2O*	NR	NR	NR	NR	No delayed hemorrhage, rapid ICP normalization
Malek & Heilman (109) [21]	USA	FS	NA	4	NA	NA	Feasibility study using 4 cadaver heads Proof-of-concept of a novel endovascular approach to the CPA cistern for CSF diversion using a low-profile minimally invasive transvenous transfemoral delivery system						
Malek & Heilman (E-020) [22]	USA	FS	NA	NA	NA	NA	Describes engineering and in vitro testing of novel endovascular CSF shunt mimicking arachnoid granulations						
Malek et al. (Abstract 12) [23]	USA, Argentina	CR	IIH	1	0	50	Successful deployment, rapid headache relief, reduced CSF pressure, improvement of the prominent subarachnoid space at 30 days. Repeat lumbar puncture revealed a lowed opening pressure of 20 cmH2O, MRI-confirmed patency of biometric valve						

*CPA* cerebellopontine angle; *CR* case report; *CSF* cerebrospinal fluid; *EVD* external ventricular drain; *FS* feasibility study; *ICP* intracranial pressure; *IIH* idiopathic intracranial hypertension; *iNPH* idiopathic normal pressure hydrocephalus; *MOCA* Montreal Cognitive Assessment; *mRS* modified Rankin Scale; *NA* not applicable; *NBSS* Neurogenic Bladder Symptom Score; *NPH* normal pressure hydrocephalus; *NR* not reported; *PO* prospective observational; *post-aSAH* post-aneurysmal subarachnoid hemorrhage; *SAE* serious adverse event; *SD* standard deviation; *TUG* Timed Up and Go test

### Preclinical and Anatomical Studies

Early hypotheses were based on the feasibility of using natural venous drainage pathways, particularly the inferior petrosal sinus (IPS), to redirect CSF from the cerebellopontine angle (CPA) cistern to the internal jugular vein [10]. In 2018, Malek et al. developed a catheter-based endovascular system to deliver a shunt device through the IPS into the CPA cistern, mimicking the function of arachnoid granulations [21]. The system uses a stent-anchor and flat rail wire to guide a dual-lumen catheter with a retractable needle that can penetrate the dura and deploy the shunt [21].

This technique was successfully tested on four human cadaver heads, demonstrating precise transdural access and correct placement of the shunt using imaging guidance [13].

Preclinical evaluation of this approach in animal models was performed by Benatti et al. using eight ovine subjects [24]. The study demonstrated the feasibility and safety of catheterizing the CPA cistern via a transvascular approach and showed potential for central nervous system distribution of therapeutic agents [24]. Anatomical MRI studies in adults [10] (*n* = 36) and morphometric studies in 100 children [14] (*n* = 100) confirmed that the anatomical corridors required for transvascular CSF shunt implantation are accessible and consistent across age groups.

### Development of the EShunt® System

The development of the endovascular eShunt® was first conceptualized as a biomimetic transdural CSF shunting system designed to replicate the physiological properties of arachnoid granulations [13]. Initial studies by Malek and colleagues detailed the mechanical properties of this valve-based system and its ability to prevent siphoning [13].

### Technical and Procedural Details

The eShunt® System is a miniature, valved, endovascular CSF shunt designed to mimic the physiological function of arachnoid granulations by facilitating CSF resorption into the intracranial venous system [15]. It is implanted via a minimally invasive, percutaneous, transfemoral venous approach targeting the IPS and the CPA cistern [15]. The device is 3 cm in length and consists of a biomimetic slit valve that regulates CSF flow based on pressure gradients across the dura, ensuring one-way drainage from the subarachnoid space into the internal jugular venous system [15].

Implantation involves preprocedural imaging with gadolinium-enhanced MRI and cone-beam computer tomography (CT) to map the IPS and surrounding vascular anatomy, enabling virtual simulation and planning of the transdural trajectory [15]. A two-step proprietary delivery system is used: first, an exchange-length flat-wire anchor is placed into the cavernous sinus and IPS junction to serve as a guide rail [15]. The eShunt® delivery catheter is then navigated to the predefined site using 3D roadmapping [15]. Under fluoroscopic guidance, a needle at the catheter tip is unsheathed and used to penetrate the dural wall in a controlled manner, establishing access to the CPA cistern [15]. The shunt is deployed with its distal malecot tip residing within the cistern and the proximal valved end draining into the internal jugular vein [15].

The valve mechanism is designed to allow pressure-dependent flow of CSF into the venous system while preventing retrograde blood reflux during transient increases in venous pressure, such as during coughing or positional changes [15]. The implant is engineered to provide a physiologic flow rate of approximately 10 mL/hour at a pressure differential of ≤ 8 mm Hg [15]. Total procedural time is typically around 125 min, with device-specific deployment taking approximately 50 min [15].

In the first-in-human case reported by Lylyk et al. [12], the procedure resulted in immediate and sustained reduction of intracranial pressure (ICP) from 38 to < 20 cmH_2_O within 90 min post-deployment. Neuroimaging confirmed stable positioning of the implant, normalization of ventricular size, and absence of procedural complications or overdrainage [12].

### Current Indications

To date, the primary indication for eShunt® implantation is communicating hydrocephalus, primarily patients with idiopathic normal pressure hydrocephalus (iNPH), who meet clinical and morphological criteria and are considered eligible for traditional CSF diversion procedures.

Inclusion criteria of ongoing trials include the presence of at least two components of the Hakim triad (gait disturbance, cognitive impairment, and urinary incontinence), radiographic confirmation of ventricular enlargement (Evan’s Index > 0.3), a positive response to a large-volume lumbar tap test as assessed by improved performance on the Timed Up and Go (TUG) test, and a baseline Montreal Cognitive Assessment (MoCA) score ≥ 12. Eligible patients must also have CSF opening pressures ≥ 8 cmH_2_O and no evidence of obstructive or non-communicating hydrocephalus. Neuroimaging (CT and MRI) is used to confirm the absence of contraindications such as mass lesions, venous anomalies, or anatomic barriers that would preclude safe implantation.

Anatomical eligibility is particularly relevant to the feasibility of transvascular deployment via the IPS and CPA cistern. Morphometric studies have demonstrated that a majority of adults—and even a significant proportion of pediatric patients over one year of age—exhibit IPS dimensions and cisternal depth adequate for safe delivery of the device. While the primary indication remains adult patients with iNPH, investigational use has extended to populations with CSF circulatory disorders following aneurysmal subarachnoid hemorrhage and idiopathic intracranial hypertension (IIH).

### First-in-Human Studies

The case reports by Lylyk et al. reported the first successful human applications of the eShunt® system [12]. In a patient with post-subarachnoid hemorrhage (SAH) hydrocephalus, they demonstrated that eShunt® implantation resulted in rapid reduction of intracranial pressure (ICP) and resolution of ventriculomegaly [12].

### Clinical Studies

Five prospective clinical trials have been initiated to evaluate the safety and efficacy of the eShunt® system in patients with NPH. The design characteristics, inclusion criteria and primary endpoints of the currently ongoing clinical studies are presented in Table 3. The ETCHES I trial (NCT04758611) is the first, prospective, single-center pilot study from Argentina evaluating the safety and efficacy of endovascular CSF in 30 adult patients with post-aneurysmal SAH and communicating hydrocephalus who failed external ventricular drain (EVD) clamping. Primary endpoints include ICP at 36–48 h and long-term outcomes at 6, 12, and 24 months. The trial began in October 2020 and is expected to complete in December 2027.

**Table 3:** Characteristics of completed or ongoing clinical studies sorted by their date of start.

Trial ID	Country	Number of centers	Design	Number of Participants	Inclusion Criteria	Key Endpoints	Status	Date of Start	Expected primary Completion	Expected study completion	Findings (if available)
NCT04758611 (ETCHES I)	Argentina	1	Prospective, single-center, open label, single-arm pilot study	30	Age ≥ 18 yrs; post-aneurysmal SAH with EVD in place and failed EVD clamp; no signs of vasospasm, communicating hydrocephalus; CT/MRI-suitable anatomy; no obstructive hydrocephalus/infection	ICP at 36–48 h; conversion to conventional VPS; neurological evaluations and imaging at 180 days, 12 and 24 months	Active, recruiting	2020-10-01	2025-12-01	2027-12-01	NA
NCT05250505	Argentina	1	Prospective, non-randomized, open-label, single-center, pilot study	30	Age 65–85 yrs, pts w/NPH eligible for traditional CSF shunt, clinical presentation w/≥ 2 symptoms of the hakim trias together with Evans’ Index > 0.3, improvement in TUG Test after spinal tap test, CSF opening pressure ≥ 8 cmH2O and baseline MoCA ≥ 12, ≥ 20%, CT- and MRI-confirmed anatomy suitable for eShunt® implantation	Change in gait (measured by TUG), cognitive ability (measured by MoCA), urinary tract sx, mRS, SAE at 90, 180 and 365 days	Recruiting	2022-03-10	2025-07 (estimated)	2027-04 (estimated)	Pending
NCT05232838	USA	11	Prospective, non-randomized, open-label, multi-center pilot study	50	Age 65–85 yrs, pts w/NPH eligible for traditional CSF shunt, clinical presentation w/≥ 2 symptoms of the hakim trias together with Evans’ Index > 0.3, improvement in TUG Test after spinal tap test, CSF opening pressure ≥ 8 cmH2O and baseline MoCA ≥ 12, ≥ 20%, CT- and MRI-confirmed anatomy suitable for eShunt® implantation	Change in gait (measured by TUG), cognitive ability (measured by MoCA), urinary tract sx, mRS, SAE at 90, 180 and 365 days	Active, not recruiting	2022-04-20	2025-06-15	2030-06-30 (estimated)	Interim results of 30 patients (March 2024):97% (29/30) improved; no device- or procedure-related SAEs; mean hospital stay 1.3 days
NCT05501002	USA	11	Prospective, non-randomized, open-label, multi-center pilot study	4	Age ≥ 21 yrs; post-aneurysmal SAH with EVD in place ≥ 7 days and failed EVD clamp; communicating hydrocephalus; CT/MRI-suitable anatomy; no obstructive hydrocephalus/infection	ICP at 48 h; neurological evaluations and imaging at 30, 60, 90, 180, 365 days; annual follow-up	Terminated	2022-09-15	2023-09-15	2023-09-15	NA
NCT06498960 (STRIDE)	International	15	Prospective, multi-center, randomized, controlled trial	230	Age > 60 yrs w/NPH eligible for traditional CSF shunt, hx of gait impairment w/duration ≥ 3 months, clinical presentation w/≥ 2 symptoms of the hakim trias together with Evans’ Index > 0.3, improvement in TUG Test after spinal tap test, CSF opening pressure ≥ 8 cmH2O and baseline MoCA ≥ 12, ≥ 20%, CT- and MRI-confirmed anatomy suitable for eShunt® implantation	Change in gait impairment at 6‑months measured by TUG, SAE at 6 months	Recruiting	2024-11-26	2026-07-01 (estimated)	2031-01-01 (estimated)	Pending

*CSF* cerebrospinal fluid; *CT* computed tomography; *EVD* external ventricular drain; *ICP* intracranial pressure; *MoCA* Montreal Cognitive Assessment; *MRI* magnetic resonance imaging; *mRS* modified Rankin Scale; *NPH* normal pressure hydrocephalus; *SAE* serious adverse event; *SAH* subarachnoid hemorrhage; *TUG* Timed Up and Go test; *VPS* ventriculoperitoneal shunt

NCT05501002 was a U.S.-based, prospective, open-label pilot study evaluating the eShunt® system in adult patients with communicating hydrocephalus following aneurysmal SAH. The trial enrolled four patients across two centers but was terminated early, with data collection continuing through long-term follow-up. Key endpoints included intracranial pressure at 48 h and neurological and radiographic outcomes up to one year.

The U.S.-based pilot study (NCT05232838), is a non-randomized, open-label, multi-center trial that enrolled 50 patients aged 65–85 years with a clinical diagnosis of NPH. First interim results of the first 30 patients were presented in March 2024 and demonstrated an improvement of clinical NPH-typical symptoms in 97% (29/30) patients without any device- or procedure-related serious adverse events (SAEs), and a mean hospital stay of 1.3 days. While patient recruitment has concluded, the one-year follow-up is still collected, with the final study completion estimated to be in June 2030.

A parallel single-center pilot study in Argentina (NCT05250505) is currently recruiting patients with a planned number of 30 included patients. The design, inclusion criteria and endpoints correspond to the aforementioned U.S. trial. The final completion is estimated by April 2027.

In parallel, the STRIDE-trial is the first international, multicenter RCT (NCT06498960) that compares the eShunt® system with traditional ventriculoperitoneal shunting. This trial aims to enroll 230 patients in 15 centers. The primary endpoint was defined as gait impairment at 6 months measured by TUG test. In addition, further secondary outcomes and SAEs are collected. The STRIDE trial began enrollment in late 2024, with primary completion expected in July 2026 and full study completion projected for January 2031.

### Early Results

To date, no original peer-reviewed publication has reported outcome data from a patient cohort treated with the eShunt® system. In two studies on cases with post-aSAH hydrocephalus (Lylyk et al. [16] (*n* = 7) and Lylyk et al. [20] (*n* = 9)), EVD removal within 36–48 h post-insertion was successful in all treated patients (7/7 and 9/9, respectively). In both cohorts, ICP decreased markedly—from a baseline of 33.4 to 13.0 cmH_2_O at 1 h, and further to 9.0 cmH_2_O at 36 h [16], and from 34.4 to 10.8 cmH_2_O at 1 h and 10.9 cmH_2_O at 36 h [20]. No delayed hemorrhage or device-related adverse events were observed [16, 20].

In two studies on patients with iNPH (Lylyk et al. [17] (*n* = 11) and Lylyk et al. [9] (*n* = 19)), improvement in the Normal Pressure Hydrocephalus Symptom Scale (NBSS) was noted at 30, 90, and 180 days, with rates of 35.4%, 24.8%, and 32.8%, respectively, [9, 17] and 32.1%, 33.3%, and 39.0%, respectively [9]. At one year follow-up, TUG test performance improved by 35.7% while mean MoCA scores improved by +1.6 points, while NBSS score improved by −3.6s [19]. Fifteen of 16 patients (93.8%) demonstrated clinical improvement in at least one symptom of the Hakim triad [9]. In a study evaluating one-year safety of the eShunt® implant in 14 patients with communicating hydrocephalus (9 with NPH, 4 post-aSAH, and 1 with IIH), no cases of radiologic or symptomatic overdrainage, hemorrhage, or implant migration were observed, and modified Rankin scores (mRS) were either stable or improved compared to baseline [18].

## Discussion

Ventriculoperitoneal shunting represents the current standard treatment for symptomatic communicating hydrocephalus, however, it is associated with rates of shunt infection of around 10%, and shunt failure between from 21% to 42% within the first year after placement [6–8]. Endovascular shunting has been proposed as a new and minimally invasive approach to improve CSF drainage. This systematic literature review provides the first systematic synthesis of the technical development, procedural aspects, current clinical applications, and early results of endovascular CSF devices.

The eShunt® system represents a novel method to mimic arachnoid granulation function and allow CSF absorption into the internal jugular system. Preclinical studies in cadaveric models have demonstrated the anatomical feasibility of the approach through the IPS and showed that CPA cistern provides a stable and reproducible route for transdural shunt deployment.

Currently available outcome data—mainly from conference abstracts—demonstrate a rapid reduction in ICP, resolution of ventricular dilatation on imaging and a favorable safety profile without radiologic or signs of overdrainage, hemorrhage, or implant migration. Lylyk et al. [25] found that eShunt® implantation led to a clinical improvement in at least one symptom of the Hakim triad in 93.8% of patients. In comparison, a systematic review and meta-analysis of 33 studies involving 2461 patients with iNPH reported that traditional VPS led to clinical improvement in approximately 75% of patients [19].

At one-year follow-up after eShunt® insertion, TUG performance improved by 35.7%, mean MoCA scores improved by +1.6 points, while NBSS performance improved by −3.6s [10]. These values are largely comparable to previously reported rates of improvement in TUG test and MOCA after VPS. In a systematic review analyzing the time course of symptoms after VPS in patients with iNPH, improvements in MMSE scores, studies with twelve-month follow-up duration showed improvements between 0.3 and 2 points [26]. Sundström et al. reported a median improvement in the TUG test of ~5s (~26%) at unspecified follow-up [27]. In an analysis of 193 patients with iNPH, the mean MoCA score improved from 21.0 ± 5.0 to 22.6 ± 5.5 (+1.6 ± 0.5) at 8–12 months after VPS [28].

To date, five prospective clinical trials have been initiated. Interim results from the U.S. pilot study (NCT05232838) suggest an excellent safety profile, a high procedural success rate and short hospital stays, with 97% of patients achieving clinical improvement without any device- or procedure-related serious adverse events. However, NCT05501002 was terminated early—due to low enrollment according to clinicaltrials.gov—, with no official press release on the reason for termination.

Despite the promising early results of this new device, clinical translation remains at an early stage. The current body of literature on endovascular treatment of hydrocephalus remains sparse, with only a handful of published peer-reviewed original studies. The majority of included studies, case reports, letters and opinion pieces are authored or co-authored by device developers, introducing a potential bias in outcome reporting and interpretation.

Due the limited follow-up durations and standardization of outcomes, there remain unanswered questions regarding long-term durability, valve integrity, and the risk of delayed complications such as thrombosis, reflux, or shunt failure. Additionally, the physiological impact of continuous CSF diversion into the venous system—particularly under variable central venous pressures—requires further studies. While morphometric studies suggest anatomical feasibility in older children, no clinical trials have yet assessed the safety or efficacy of endovascular CSF shunting in pediatric populations. Given the amount of shunt revisions in this group, the potential for minimally invasive alternatives merits further investigation. Most importantly, comparative data with VPS will be essential for regulatory approval and broad clinical application.

From a technical standpoint, the widespread clinical adoption of endovascular CSF shunting devices demands not only a deep understanding of cerebral venous anatomy but also proficiency in imaging modalities such as cone-beam CT and 3D roadmapping. Rigorous patient selection and training standards will be crucial to reproduce the success rates and outcomes reported by current studies.

While the current body of literature focusses mainly of technical details and outcomes of eShunt®, other techniques such as choroidal artery embolization have been described. As evidence on these techniques is limited to singular case reports and cadaveric studies, these techniques warrant further investigation.

## Limitations

First, the current body of literature on endovascular shunting mainly consists of preclinical studies, case reports, and conference abstracts. The absence of published RCTs and high-quality comparative studies substantially limits the ability to draw robust conclusions about clinical efficacy, safety, and generalizability. The majority of included reports are authored by one single group of investigators, introducing a potential publication and reporting bias. Furthermore, the available human data are limited to highly selected patients from early feasibility trials or single-case experiences, which may not reflect outcomes in broader or more complex clinical populations. Notably, anatomical eligibility for endovascular shunting represents a critical limitation. Morphometric analysis by Colasurdo et al. indicated that the eShunt® procedure may be feasible in only approximately 67% of patients due to anatomical constraints of the inferior petrosal sinus and cerebellopontine angle cistern [14]. The published literature lacks detailed reporting of exclusion rates attributable to anatomical limitations, precluding accurate estimation of the proportion of patients eligible for eShunt® implantation in clinical practice. The lack of standardization in outcome measures and limited follow-up durations further restrict the interpretation of outcome data. Although a comprehensive search strategy was employed across multiple databases and supplemented by individual literature search, it is possible that relevant unpublished data, non-English language publications, or studies indexed in less common repositories were missed. As a result, the present review may not fully capture all emerging literature in the field. While the systematic literature review methodology allows for mapping of the existing literature and identification of gaps, it does not include critical appraisal of study quality or risk of bias, which limits the interpretability of the findings.

## Conclusion

Endovascular CSF diversion represents a minimally invasive alternative to traditional shunting techniques for communicating hydrocephalus. Preliminary data indicates that the scope of indications may further extend to normal pressure hydrocephalus or idiopathic intracranial hypertension. While early results demonstrate technical feasibility and encouraging safety profiles, long-term data from ongoing clinical trials are critical to establish its role in standard neurosurgical and neurointerventional practice.

## Supplementary Information

ESM1: Supplementary material 1
