# Supplementary material for: Endovascular Treatment of Hydrocephalus: A Systematic Literature Review
Source: Clin Neuroradiol. 2026 Jan 21;36(2):309–20. doi: 10.1007/s00062-026-01614-y (PMC13319876; doi:10.1007/s00062-026-01614-y)
Supplement: Supplementary file 1 — ESM1: Supplementary material 1 [file 62_2026_1614_MOESM1_ESM.docx]

**Supplement**

**Supplementary Table 1: Search strategy**

| **Database** | **Search Strategy** | **Results** |
| --- | --- | --- |
| PubMed | (endovascular[Title/Abstract]) AND (shunt[Title/Abstract] OR hydrocephalus[Title/Abstract]) | 1,450 |
| Embase | 'endovascular':ab,ti AND ('shunt':ab,ti OR 'hydrocephalus':ab,ti) | 2,072 |
| Scopus | TITLE-ABS(endovascular) AND (TITLE-ABS(shunt) OR TITLE-ABS(hydrocephalus)) | 1,797 |

**Supplementary Table 2:** Reasons for exclusion.

| **Rank** | **Reason for Exclusion** | **Count** | **Percentage** |
| --- | --- | --- | --- |
| 1 | Evaluation of shunting techniques (except for eShunt) in aneurysms/SAH | 790 | 28.6% |
| 2 | Evaluation of shunting techniques (except for eShunt) in arteriovenous fistulas | 644 | 23.3% |
| 3 | Shunt mechanisms/procedures in liver diseases | 495 | 17.9% |
| 4 | Not about shunting techniques (e.g. studies on MMA embolization, endovascular aneurysm treatment, other neurointerventional techniques) | 467 | 16.9% |
| 5 | Shunt mechanisms/procedures in pulmonary diseases | 134 | 4.9% |
| 6 | Shunt mechanisms/procedures in cardiac diseases | 72 | 2.6% |
| 7 | Conference proceedings with only abstract/title, insufficient information, or full text unavailable | 55 | 2.0% |
| 8 | Shunt mechanisms/procedures in peripheral vascular diseases | 51 | 1.8% |
| 9 | Evaluation of shunting techniques (except for eShunt) in venous sinus stenosis | 18 | 0.7% |
| 10 | Evaluation of shunting techniques (except for eShunt) in spinal vascular diseases | 17 | 0.6% |
| 11 | Evaluation of shunting techniques in general interventional radiology | 13 | 0.5% |
| 12 | Shunt mechanisms/procedures in other vascular diseases | 4 | 0.1% |
| 13 | Opinion piece/narrative review | 1 | <0.1% |
